# Changes in Eating Behaviour during SARS-CoV-2 Pandemic among the Inhabitants of Five European Countries

**DOI:** 10.3390/foods10071624

**Published:** 2021-07-13

**Authors:** Paulina Górska, Ilona Górna, Izabela Miechowicz, Juliusz Przysławski

**Affiliations:** 1 Department of Bromatology, Poznań University of Medical Sciences, 60-354 Poznan, Poland; igorna@ump.edu.pl (I.G.); jotespe@ump.edu.pl (J.P.); 2 Department of Computer Science and Statistics, Poznań University of Medical Sciences, 60-806 Poznan, Poland; iza@ump.edu.pl

**Keywords:** eating behaviour, consumer preference, COVID-19

## Abstract

Psychological factors and restrictions imposed due to the pandemic may influence eating behaviours and physical activity. With the above thesis in mind, questionnaire-based surveys were conducted amongst residents of five European countries: Poland, Italy, Spain, Portugal and Great Britain (England and Scotland). A specially devised, structured questionnaire was used to conduct anonymous internet surveys between 28 April and 16 July 2020. It contained questions pertaining to sociodemographic data, eating behaviours, the impact of the pandemic on the diet and physical activity. The questionnaire was made available to internet users in Poland, Italy, Spain, Great Britain (England and Scotland), and Portugal. The questionnaire was translated by native speakers into five languages: Polish, English, Spanish, Italian and Portuguese. Survey results were then analysed using StatSoft’s Statistica v. 13 software and Cytel’s StatXact v. 9.0.0. Age was the parameter that impacted changing eating behaviours to the largest extent during the pandemic. It was also found that during the pandemic, regular consumption of meals was most dependent on various factors. The negative impact of the pandemic within this scope was most profound amongst women, city residents regardless of gender and people over 35 years of age. A change in the frequency of consumption of selected product groups during the pandemic was also observed. Reduced consumption of meat and fish was identified. Especially among people under 35 living in Portugal, almost half—45.5% (*p* = 0.0210) declared lower consumption of meat, and more than half—54.5% (*p* = 0.011) reported lower consumption of fish. An analysis of the obtained results also showed an increase in the consumption of products with lower nutritional values, particularly amongst people under 35 years of age and also amongst residents of Great Britain (regardless of age). Moreover, the results showed that the pandemic may have had an impact on the weight reduction diet. A negative impact was declared by 16.5% of people, compared to 9.7% who said that the pandemic facilitated the use of the weight reduction diet (*p* = 0.006). The results of our survey also showed a decrease in the level of physical activity among people over 35 living in Poland (69.6%, *p* = 0.0497) and people living in Portuguese cities (72.73%, *p* = 0.0245). Our survey results showed that the impact of the pandemic on eating behaviours was particularly profound when it came to meal consumption regularity. Changes to the consumption of products with lower nutritional values, which may decrease immunity, have also been found during the pandemic. Our results showed that the problem associated with consuming products with lower nutritional values was particularly evident amongst people under 35. Considering the global character of SARS-CoV-2 transmission, further research is necessary to determine its impact on the diet, nutritional status and physical activity.

## 1. Introduction

Towards the end of 2019, several pneumonia cases of unknown aetiology [1,2] were reported in Wuhan, the capital of the Chinese Hubei Province. The virus causes the onset of a disease, which the World Health Organisation named “coronavirus 2019” (COVID-19) [3]. Approximately two months after the CDC identified the new disease, the World Health Organisation characterised the spread of the disease as a pandemic [4].

The scale with which the virus is able to spread is exceptional. This has forced the governments of many countries to freeze many sectors of the economy, social and cultural life, which is why it is so important to find an answer to how such an unusual situation affects our eating behaviours and physical activity required to stay healthy. Moreover, because we cannot forecast when the pandemic will end, this might profoundly impact public health.

Social isolation is one of the most effective means used to control the pandemic [5]. However, despite its benefits in curbing the spread of the virus, it also has negative consequences. Data acquired during the SARS-CoV epidemic have shown that quarantine is associated with a higher risk of mental disorders, exhaustion, anger, sleep problems and the occurrence of depression and post-traumatic stress symptoms [6,7,8]. Psychological factors may influence eating behaviours. In a survey conducted during the SARS-CoV-2 pandemic, 42.7% of the respondents indicated heightened anxiety as the leading cause for changing eating behaviours [9]. Stress may contribute to increased consumption of products with lower nutritional values, amongst other behaviours [10]. Restrictions imposed due to the pandemic by governments in their respective countries may also influence eating behaviours by altering the individual economic situations [9].

Surveys carried out amongst Italian residents indicated that during the pandemic, approximately 21.2% of the respondents consumed more fresh fruit and vegetables and less alcohol (36.8% of the respondents). 19.5% of the respondents reported an increase in body weight. Increased consumption was particularly evident for products such as ice creams and desserts (42.5%), as well as savoury snacks (23.5%) [9]. Other surveys found that the frequency of snacking between meals and the number of main meals also increased [11]. Reduced levels of physical activity were also noted [12].

The aim of the study was to assess changes in eating behaviour and physical activity during a pandemic in a group of people living in various European countries. Apart from identifying differences between the various countries, we wanted to check whether other factors such as place of residence (city or village), gender and age are significant. Compared to other studies that have looked at changes in eating behaviour during a pandemic, the aim of the study was also to see how the pandemic affects the weight reduction diet. Moreover, the design of the questionnaire allowed for a subjective assessment of changes in eating behaviour before and during the pandemic, not only for the assessment of eating behaviour during it.

## 2. Materials and Methods

### 2.1. Study Design and Participants

A specially devised, structured and anonymous internet questionnaire was used as the research tool. From the research point of view, the respondents who answered the questionnaire did not have to be nationals of the country in question but had to reside there during the pandemic. Only people older than 18 took part in the survey. Participants aged <18 years old were excluded. Age was verified using a question in the questionnaire. Data were collected via an online survey running over three months (April–July 2020). Respondents were recruited on social networks through several groups on Facebook (for example, on groups that bring together students and people living in various places in Poland, Italy, Portugal, Great Britain—Scotland and England, Spain) and through posts on LinkedIn, Instagram and Twitter. Native speakers translated the questionnaire into five languages: Polish, English, Spanish, Italian and Portuguese. The internet survey was conducted in agreement with the national and international regulations and the Declaration of Helsinki. All participants were informed about the objectives and requirements of the study. They filled out the questionnaire by connecting to Google Forms, and then the questionnaires were downloaded as Microsoft Excel sheets with a guarantee of anonymity. The questionnaire did not include questions about personal data, and all questionnaires were downloaded as a Microsoft Excel sheet. The Poznan University of Medical Sciences Bioethical Committee declared that no ethical clearance was needed.

### 2.2. Measures

The questionnaire used for the survey contained questions pertaining to sociodemographic data (gender, age, place of residence), eating behaviours, the impact of the pandemic on the diet and physical activity. Respondents were asked to make a subjective assessment of changes by comparing the period before the pandemic to the period during the pandemic.

#### 2.2.1. Meal Consumption Regularity, Eating Behaviours, Frequency of Food Consumption

The questionnaire included questions on how regularly meals were consumed and on the frequency of consumption of selected product groups: milk and dairy products, cereal products, eggs, meat and meat-based products, fish, fats (vegetable oils; butter; margarine; cream; other animal fats, e.g., lard; mayonnaise and dressings e.g., salad dressings), vegetables, fruit and pulses. Respondents were also asked whether the pandemic impacted consumption levels of products with lower nutritional values: sweets, savoury snacks, fizzy soft drinks, energy drinks, fast foods and instant meals. The questionnaire also included a question on changes to the frequency of alcohol consumption. The questionnaire was developed based on the nutritional recommendations published by the WHO and the CDC during the pandemic [13,14].

#### 2.2.2. Impact of the Pandemic on the Weight Reduction Diet

One of the research objectives was to find an answer to whether the pandemic had affected the weight reduction diet. Respondents were asked to make a subjective comparison of the ease of adherence to dietary rules before and during the pandemic. In a closed question, respondents could indicate a number of factors that made an impact. It was assumed that a positive impact might be the result of having more time to prepare correctly balanced meals and/or to find out more about healthy nutrition, improved conditions for eating meals at regular times, and/or no temptation and pressure within the scope of consuming low nutritional values meals during family/social meetings. On the other hand, a negative impact may be the result of deteriorated conditions for eating meals at regular times, less time to prepare well-balanced meals, increased desire to eat products with lower nutritional values (e.g., sweets, savoury snacks) and/or more difficult access to medical facilities and dieticians.

#### 2.2.3. Physical Activity

Individuals taking part in the survey were asked whether their physical activity level during the pandemic decreased, increased or had not changed.

### 2.3. Statistical Analyses

Survey results were then analysed using StatSoft’s Statistica v. 13 software and Cytel’s StatXact v. 9.0.0. The Shapiro–Wilk test was used to test the distribution of variables for normality. To compare two groups of normally distributed variables with the same variance, the Student’s t-test was used for unrelated individuals and the Mann–Whitney test was used where the variables were not normally distributed. To compare a larger number of groups (between countries), as the normality criteria were not satisfied, the Kruskal–Wallis test followed by Dunn’s Multiple Comparison Test were applied. To test for dependencies among categorical variables, the Chi-squared test for independence, Fisher’s exact test or the Fisher–Freeman–Halton test were used. The statistical power of the study was between 0.66 and 0.98. Therefore, an α = 0.05 significance level was adopted. Results were considered to be statistically significant for *p* < 0.05.

## 3. Results

### 3.1. Demographic Characteristics

A total of 279 people took part in the survey. Participant characteristics can be seen in Table 1.

### 3.2. Meal Consumption Regularity

**Figure 1:**
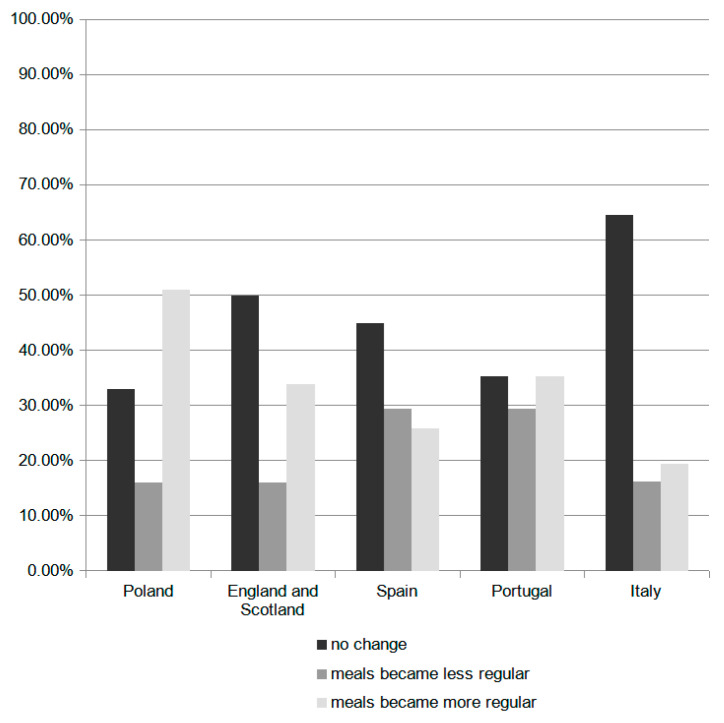
Meal consumption regularity during the pandemic per country.

It was observed that there was a statistically significant dependence (*p* = 0.0045) between meal consumption regularity during the pandemic and the country of residence (Figure 1).

Within the group comprising people residing in Poland, it was observed that the pandemic contributed to improved meal consumption regularity for more than half (56.1%) of the women (*p* = 0.0262). Concerning the place of residence, a statistically significant dependence (*p* < 0.001) was found for people residing in Portugal, where residents of cities were more likely to declare that the pandemic contributed to decreased meal consumption regularity. On the other hand, more than half of those living in the country (66.7%) stated that their meals became more regular. The respondents’ age was also a significant factor within the scope of meal consumption regularity. That relationship is shown in Table 2.

### 3.3. Eating Behaviours

Great Britain (England and Scotland) residents were most likely to declare increased consumption of products with lower nutritional values. As compared to respondents from other countries, they were more likely to state that during the pandemic, they ate more savoury snacks (25.9%), fast food and instant meals (12.3%) and consumed more energy drinks (7.4%). Furthermore, 25.9% of respondents from Great Britain indicated increased alcohol consumption during the pandemic. Increased consumption of sweets was most frequently indicated by residents of Portugal (41.2%). In comparison, residents of Spain showed increased consumption of fizzy soft drinks (13.8%) and fried dishes (13.8%). At the same time, residents of Poland (28%) were most likely to declare that they paid more attention to the principles of healthy eating. Assuming that the pandemic could have not affected the respondents’ eating behaviours, the questionnaire did include such an answer. Residents of Portugal were most likely to select that answer (38.2%). These results were statistically significant (*p* < 0.05).

A statistically significant dependence (*p* < 0.05) between gender, the place of residence (village or city) and eating behaviours during the pandemic was not found in any of the countries in question. However, such dependence was found for age in a group of people living in Poland. Participants under 35 years of age more often declared that during the pandemic, they increased their consumption of savoury snacks (24.68%). In the group of people above 35 years of age, 4.35% reported increased consumption of savoury snacks (*p* = 0.0375).

### 3.4. Frequency of Food Consumption

Respondents were asked how the frequency of consumption of given product groups changed during the pandemic. It was observed that among the respondents from all the countries, more people declared an increase in the consumption of cereal products (with the highest percentage recorded in the case of inhabitants of Portugal—32.25%, *p* = 0.4784). In Portugal, people up to 35 years of age were more likely to declare a higher consumption of cereal products than people over 35 (*p* = 0.0339). No statistically significant dependence between the frequency of consumption and gender, age or place of residence was found (*p* > 0.05).

In the case of milk and dairy products, the inhabitants of Poland (28.00%) and Portugal (23.53%) more often declared increased consumption of this type of product. In contrast, in Spain, a more significant percentage of respondents indicated that their consumption decreased (25.86%, *p* = 0.08959). Further analysis showed increased milk and dairy product consumption among male respondents from Great Britain—England, and Scotland (*p* = 0.0045). Age was another factor for which a statistically significant relationship was identified (*p* = 0.0269)—23.1% of people up to 35 years of age indicated higher consumption of milk and dairy products while 6.7% of respondents above 35 years of age indicated that answer. The situation was similar in Spain (*p* = 0.0161) and Italy (*p* = 0.0370), where people up to 35 years of age declared higher consumption of this product group more often.

It was observed that among the respondents from all the countries, more people declared an increase in the consumption of eggs (with the highest percentage recorded in the case of inhabitants of Portugal—32.29%, *p* = 0.0756). No statistically significant dependence between the frequency of consumption and gender, age or place of residence was found (*p* > 0.05).

Frequency of consumption of meat and meat products was also analysed. It was observed that among the respondents from all the countries, more people declared a decrease in the consumption of meat and meat products (with the highest percentage recorded in the case of inhabitants of Spain—31.03%, *p* = 0.4837). Moreover, it was shown that people up to 35 years of age who live in Portugal were more likely to declare decreased meat and meat product consumption during the pandemic—45.5% of respondents in this age group declared decreased consumption (*p* = 0.0210).

In the case of fish, respondents from all countries more often declared a reduction in their consumption (with the highest percentage in Italy—29.03%, *p* = 0.5217). Further analysis of the results showed a statistically significant relationship between the age of those living in Portugal and the frequency of fish consumption during the pandemic (*p* = 0.0011). More than half (54.5%) of people up to 35 years of age declared that their consumption of those products decreased.

The frequency of consumption of fats was also analysed. The increase in their consumption during the pandemic was most often declared by the inhabitants of Italy (19.35%), while an overall decrease was reported by the participants from Spain (27.59%, *p* = 0.2209). In Portugal (*p* = 0.0011) and Italy (*p* = 0.0105), the frequency was found to be age-dependent. In both countries, people up to 35 years old increased their consumption during the pandemic.

It was observed that among the respondents from all the countries, more people declared an increase in the consumption of vegetables (with the highest percentage recorded in the case of inhabitants of Spain—43.10%, *p* = 0.1189) and fruits (with the highest percentage recorded in the case of inhabitants of Spain—39.66%, *p* = 0.2244). For vegetables and fruits, significant differences were observed between age and frequency of consumption of vegetables (*p* = 0.0368) and fruit (*p* = 0.0012) during the pandemic. This consumption increased in the over 35 age group for both vegetables and fruit. Whereas in Portugal, respondents in the under 35 age group were more likely to declare increased consumption of fruit (*p* = 0.0492).

Pulses constitute a significant group of foodstuffs. The increase in their consumption during the pandemic was most often declared by the inhabitants of Spain (25.86%), while a decrease was reported by the partipants from Great Britain (21.43%, *p* = 0.1682). It was shown that in the group of respondents from Poland, 22.5% of women increased their consumption of pulses during the pandemic. Correspondingly, 11.1% of men declared higher consumption (*p* = 0.0178).

### 3.5. Impact of the Pandemic on the Weight Reduction Diet

It was observed that the pandemic might have an impact on the weight reduction diet. A negative impact was declared by 16.5% of people, compared to 9.7% who said that the pandemic facilitated the use of the weight reduction diet (*p* = 0.006). Individuals who declared a negative impact of the pandemic were most likely to indicate increased craving for unhealthy products (69.6%) as the leading cause. Out of the factors that had the most significant positive impact on their diets during the pandemic, respondents were most likely to indicate having more time to prepare healthy meals (74.1%) as the leading factor. The impact of the pandemic on the diets of residents of various countries is shown in Table 3.

Further analysis showed that for respondents from Portugal, there was a dependence between the place of residence and the impact of the pandemic on diet (*p* = 0.0055). Half of those living in cities declared it has a negative impact. Furthermore, respondents were most likely to indicate increased craving for unhealthy products as the leading cause for changes in diet (*p* = 0.0356). Furthermore, the obtained results made it possible to conclude that the impact of the pandemic on the weight reduction diet may also be age-dependent. This is shown in Table 4.

### 3.6. Physical Activity

It was observed that age could be a significant factor influencing the level of physical activity. In the group of respondents living in Poland, 69.6% of people over 35 stated that their level of physical activity had decreased (*p* = 0.0497). For those living in Portugal, the place of residence was significant. People living in cities were more likely to report reduced levels of physical activity (*p* = 0.0245).

## 4. Discussion

### 4.1. Principal Findings

#### 4.1.1. Country Differences

The results of the research conducted showed that problems with adherence to the principles of a healthy diet may differ from the country. In the case of respondents from Spain and Portugal, problems with the regularity of eating meals were more frequent than in other respondents. In addition, the problem was the increase in the consumption of products such as sweets (in the case of Portuguese residents) and sweet sodas and fried foods (in the group of people living in Spain). However, the greatest problem with the consumption of low-nutrient products was observed among people from Great Britain. Compared to respondents from other countries, they more often declared an increase in consumption of savoury snacks, fast food, instant meals, energy drinks and alcohol. On the other hand, only 16.07% of respondents in Great Britain indicated that the regularity of eating meals had decreased, and 50.00% said it was unchanged. Different results were obtained among respondents from Poland, who stated that they adhered to the principles of a healthy diet more often, and the regularity of their meals had improved. The results obtained in the group of people from Poland showed that the pandemic can positively and negatively impact eating behaviour. A positive effect was also observed concerning the consumption of vegetables, fruits and legumes among the inhabitants of all countries. Similarly to the research by Rodríguez-Pérez et al. (2020), Renzo et al. (2020) and Ruiz-Roso et al. (2020), we also noted an increased consumption of vegetables and fruit and pulses (especially in Spain) [15,16,17]. This is in line with World Health Organisation (WHO) guidelines [13]. These are products with high nutritional values, characterised by a high content of nutrients that boost immunity. For fruit and vegetables, the advantage is that they can be consumed quickly. Pulses require longer preparation times, but the advantage is that they can be stored for a longer period [12]. Concerning meat and fish, we observed lower consumption (among the respondents from all the countries), as did Renzo et al. (2020) [15]. This may be due to the short shelf life and more time required to prepare them for meat and fish.

More than half of the respondents (53.8%) reported decreased physical activity during the pandemic. The inhabitants of Portugal most often declared a decrease in their levels of physical activity. Among the respondents from Portugal, negative changes were also observed in the meal consumption regularity and the frequency of consumption of sweets. This can be an important signal for health promoters in the country, as lower physical activity levels combined with poor eating habits can lead to weight gain and obesity.

Such differences show that the negative impacts of a pandemic may be different around the world. The problems with adherence to the principles of a healthy diet may differ depending on the country. One of the reasons may be that the number of cases and deaths from COVID-19 have varied from country to country, which could affect the level of stress and anxiety among residents. Moreover, when comparing changes in eating habits between individual countries, it should be considered that they may also be caused by factors not related to the pandemic. Other influences that may also be of importance here include cultural and religious factors and culinary traditions.

#### 4.1.2. Age Differences

Pursuant to the research, age was found to be the factor that impacted changing eating behaviours to the largest extent. For younger people (<35 years), the pandemic has had a positive impact on the weight reduction diet and regularity of meals and has increased the frequency of consumption of cereal products, eggs, vegetables, fruit and fats. At the same time, it contributed to increased consumption of products with reduced nutritional value and reduced consumption of fish and meat.

Renzo et al. (2020) found that quarantine contributed to increasing appetites amongst young people. This might explain why meals were consumed more often [15]. At the same time, an increased appetite can be a reason for snacking and eating unhealthy snacks. Our research results confirmed this, as they demonstrated that people under 35 consumed more savoury snacks, fast food and instant meals. A study by Ammar et al. (2020) delivered similar results. Therein, subjects over 18 years of age were more likely to be willing to eat unhealthy snacks during a pandemic and eat at night [12]. Ruiz-Roso et al. (2020) obtained different results within this scope. They found lower consumption of fast food products during quarantine amongst Spanish teenagers. This could be due to the fact that they were still under the care of older individuals [16].

When comparing two age groups: <35 years and >35 years, it should be taken into account that apart from emotional factors (related to, for example, increased levels of anxiety and stress) or those resulting from the introduced restrictions, the influence on changing eating habits could also have included a change of life situation during the pandemic. The group of people <35 years could include students who returned to their family home due to the introduction of online learning. This could impact the regularity of meals and changes in the frequency of consumption of selected groups of products due to the family home diet. On the other hand, among respondents >35 years, there could be people whose level of anxiety and stress influencing eating habits was increased due to the deterioration of the financial situation. Moreover, the eating habits in this age group may have been influenced by the provision of 24 h care for children due to the closure of kindergartens and schools. Therefore, more research is needed to help explain the causes of changes in eating habits in different age groups.

#### 4.1.3. Place of Residence (Village or City)

The study results showed that there was a dependence between the place of residence, eating behaviour and physical activity. This tendency was observed in Portugal, where compared to people living in the countryside, respondents living in cities experienced more significant difficulties in adhering to the recommendations of the weight reduction diet and regular eating. Moreover, people living in cities declared a reduction in physical activity.

One of the reasons for this may be that in places with a more significant number of inhabitants, the stress related to the risk of infection and the feeling of the restrictions introduced may be more important.

### 4.2. Study Strengths and Weaknesses

The timing of the research problem is one of the strengths of this study. As the SARS-CoV-2 pandemic began at the beginning of 2020, few studies on its impact on eating behaviours and physical activity have been published thus far. Another strength was that the research group, consisting of people from different countries, was formed when the most stringent restrictions were being introduced. Knowing the differences that exist between countries provides an opportunity for international exchange of experiences.

Another strength was using an online questionnaire supported by an eating behaviours assessment questionnaire in different language versions, making it possible to form the study group from different countries quickly. This is particularly important during the pandemic, as the situation is changing rapidly. Furthermore, such a format reduces the risk of infection resulting from direct contact.

Another strength of the research was the question on the impact of the pandemic on the weight reduction diet that was implemented before the pandemic. Continuing a particular diet is essential for maintaining proper body weight.

When analysing the weaknesses of the study, the fact that the online questionnaire has certain limitations should be taken into account. These include, among other things, difficulties with validating it and verifying the entered data and no opportunity to consult possible doubts with the investigator.

Lack of inclusion and exclusion criteria was also a weakness of the research, which could affect the results. Some people may have had to deal with eating disorders, alcohol addiction or a job that did not force them to change their lifestyle during the pandemic. The number of respondents is also a weakness—quite few and varied from country to country.

## 5. Conclusions

The survey results showed differences between countries in terms of changes in eating behaviour and levels of physical activity during the pandemic. However, it should be considered that they may have resulted from cultural, regional or culinary differences specific to a given place. Therefore, when planning further research aimed at a more detailed analysis, it should be considered. Perhaps a good solution would be to add questions to the questionnaire that consider these factors by people who know the specifics of a given place.

Despite some differences between countries, the study results showed that a worrying trend in all countries is reducing the consumption of omega-3-rich fish (even in countries like Spain and Portugal). One of the reasons for this may be their short use-by date. This is a signal for both health promotion professionals and food producers. Perhaps a way to increase consumption of these products during a pandemic would be to promote ways of preparing fish and prepared products based on them that allow them to be stored longer.

The results of our research showed that special attention should also be paid to the under 35 age group. Considering that these are people who easily use digital technologies, it is worth considering their use in promoting healthy eating and regular physical activity. For this purpose, information and communication technologies (ICT) can be used increasingly during a pandemic. The use of ICT makes it possible to analyse data (including health status, eating behaviour, physical activity) and then implement a personalised and multi-dimensional intervention. ICT can be used in various ways to promote a healthy lifestyle. For young people who often use mobile applications, it is worth considering the preparation of an application that will allow them to monitor diet and physical activity. By entering data on the meals consumed and physical activity, the user will receive personalised feedback prepared in cooperation with dietitians or personal trainers. These could include clues about where the nutritional error is being made and how to fix it. The application may also have recipes for healthy meals and suggestions for exciting forms of physical activity at home. For added value, it may be possible to calculate the nutritional value of the meals consumed. It is also worth considering introducing gamification, which is more and more often used in mobile applications. The user who performs specific tasks (e.g., performs the right amount of exercise on a given day, eats a certain amount of healthy products) receives points similar to in the game, which encourages compliance with the principles of a healthy lifestyle. The use of ICT to promote healthy eating and physical activity during a pandemic should not be limited to young people. ICT can also be used in the elderly, but some age-related restrictions should be taken into account (e.g., by introducing personalised feedback in the form of a voice) [18].

We have not seen a virus spreading on such a large scale for many years. That is why it is important to determine where the problem may lie with observing healthy eating principles and regular physical activity. Through its impact on the functioning of the immune system, amongst others, a healthy lifestyle may be an essential element in the prevention of COVID-19.

## Figures and Tables

**Table 1:** Participant characteristics.

Variables	*n* (%)
N_total_	279 (100.00)
Country	
Poland	100 (36.00)
Great Britain (England and Scotland)	56 (20.00)
Spain	58 (21.00)
Italy	31 (11.00)
Portugal Age	34 (12.00)
<35 years	160 (57.35)
>35 years	119 (42.65)
Gender	
female	226 (81.00)
male	53 (19.00)

**Table 2:** Meal consumption regularity during the pandemic acc. to age.

	Poland (*n* = 100)	England and Scotland (*n* = 56)	Spain (*n* = 58)	Portugal (*n* = 34)	Italy (*n* = 31)
Percentage of Surveyed (%)					
No change					
<35 years	22.08 *	53.85	47.22	18.18	60.00
>35 years	69.57 *	46.67	40.91	43.48	66.67
Meals became less regular					
<35 years	16.88 *	19.23	25.00	45.45	20.00
>35 years	13.04 *	13.33	36.36	21.74	14.29
Meals became more regular					
<35 years	61.04 *	26.92	27.78	36.36	20.00
>35 years	17.39 *	40.00	22.73	34.78	19.05

* Significant differences, *p*-values < 0.05.

**Table 3:** Impact of the pandemic on the weight reduction diet of residents of various countries.

	Poland	England and Scotland	Spain	Portugal	Italy	*p*
Percentage of Surveyed (%)						
Positive Impact	8.00	3.57	20.69	2.94	12.90	
Negative Impact	9.00	17.86	13.79	32.35	25.81	0.0064
No impact	18.00	25.00	17.24	11.76	25.81	
I’m not on the weight reduction diet	65.00	53.57	48.28	52.95	35.48	

**Table 4:** Impact of the pandemic on the weight reduction diet acc. to age.

	Poland	England and Scotland	Portugal	Spain	Italy
Percentage of Surveyed (%)					
<35 years					
I’m not on the weight reduction diet	66.23	73.08	45.45	55.56	60.00
Positive Impact	9.09	3.85	9.09	16.67	30.00
Negative impact	9.09	11.54	36.36	8.33	10.00
No impact	15.58	11.54	9.09	19.44	0.00
>35 years					
I’m not on the weight reduction diet	60.87	36.67	56.52	36.36	23.81
Positive Impact	4.35	3.33	0.00	27.27	4.76
Negative Impact	8.07	23.33	30.43	22.73	33.33
No impact	26.09	36.67	13.04	13.64	38.10
*p*-value	0.6629	0.0301	0.6192	0.2654	0.0068

## Data Availability

Data can be accessible upon request to corresponding author.
